# Prevalence, determinants and knowledge of antibacterial self-medication: A cross sectional study in North-eastern Tanzania

**DOI:** 10.1371/journal.pone.0206623

**Published:** 2018-10-31

**Authors:** Pius G. Horumpende, Sophia H. Said, Festo S. Mazuguni, Magreth L. Antony, Happiness H. Kumburu, Tolbert B. Sonda, Charles E. Mwanziva, Stephen E. Mshana, Blandina T. Mmbaga, Debora C. Kajeguka, Jaffu O. Chilongola

**Affiliations:** 1 Department of Microbiology, Immunology and Molecular Biology, Kilimanjaro Christian Medical University College, Moshi, Kilimanjaro, Tanzania; 2 Department of Microbiology, Immunology and Molecular Biology, Kilimanjaro Clinical Research Institute, Moshi, Kilimanjaro, Tanzania; 3 Department of Preventive Medicine and Research, Lugalo General Military Hospital, Dar es Salaam, Tanzania; 4 Department of Public Health and Research, Ifakara Health Institute, Dar es Salaam, Tanzania; 5 Department of Parasitology and Entomology, National Health Laboratory Quality Assurance and Training Centre, Dar es Salaam, Tanzania; 6 Department of Microbiology and Immunology, Catholic University of Health and Allied Sciences, Mwanza, Tanzania; 7 Department of Paediatrics and Child Health, Kilimanjaro Christian Medical Centre, Moshi, Kilimanjaro, Tanzania; Natural Environment Research Council, UNITED KINGDOM

## Abstract

Self-medication is very common especially in developing countries and is documented to be associated with many health risks including antibiotic resistance. This study investigated the prevalence, determinants and knowledge of self-medication among residents of Siha District in Tanzania. A cross-sectional study was conducted among 300 residents in a rural District of Kilimanjaro region, North-eastern Tanzania from 1^st^ to 28^th^ April 2017. A semi-structured questionnaire was used to collect information regarding drugs used, knowledge, history and reasons for antibiotic self-medication. Log—binomial regression analysis was done using STATA 13 to examine factors associated with self-medication. A slightly majority of the respondents (58%) admitted to self-medication. Antibiotics most commonly utilized were amoxycillin (43%) and an antiprotozoal drug metronidazole (10%). The most common symptoms that led to self-medication were cough (51.17%), headache/ fever/ malaria (25.57%) and diarrhoea (21.59%). The most common reasons for self-medication were emergency illness (24.00%), health facility charges (20.33%), proximity of pharmacy to home (17.00%) and no reason (16.66%). Almost all reported that self-medication is not better than seeking medical consultation, 98% can result into harmful effects and 96% can result to drug resistance. The level of self-medication in this study is comparable with findings from other studies in developing countries. Pharmacies were commonly used as the first point of medical care. There is therefore a need for educative antibiotic legislative intervention to mitigate the adverse effects of antibiotic self-medication in Siha district in Tanzania.

## Introduction

Self-medication is defined as the use of drugs to treat self-diagnosed disorders or symptoms or the intermittent or continued use of drugs for chronic or recurrent disease or symptoms without a prescription or guidance from a physician[1]. Antibiotic self-medication causes excessive antibiotic exposure to humans[2,3] and is one of the documented factors responsible for development of the currently rapidly rising public health crisis, the resistance to antibiotics[2,4–10]. A clear relationship exists between microbial resistance and amount of antibiotic use[2]. Other causes of antibiotic resistance are antibiotic overuse[4][11–15], clinicians’ over-prescription[1,16,17], a strong belief by the public in antibiotics such as hoarding and non–prescription purchase[18] and veterinary antibiotic use for prophylaxis and growth promotion[19]. Other human malpractices that have contributed to the emergence and spread of antibiotic resistance include inappropriate antibiotic use, inadequate dosing and incomplete doses[20].

Among the measures taken by a person who feels unwell in a typical developing country is the use of traditional medication alongside modern medicine[21]. The latter involves buying medicines from a pharmacy as the first point of care without necessarily seeking medical consultation[18]. Medicines can be accessed often without a prescription both in developed world[22] and in developing countries[18]. The majority of patients resort to self medication[23,24], a breeding ground for antimicrobial resistance (AMR).

The adverse effects of self-medication include incorrect self-diagnosis, delays in seeking medical advice when needed, infrequent but severe adverse reactions, dangerous drug interactions, incorrect manner of administration, incorrect dosage, incorrect choice of therapy, masking of a severe disease and a risk of dependence and abuse[25]. Many of the mentioned self-medication risks lead to AMR. This study is justified due to the fact that only a small number of studies have been done in the North Eastern zone of Tanzania on antibiotic self-medication and that self-medication and non-doctor prescription with antibiotics is associated with development of antibiotic resistance, cross-resistance, and treatment failure.

This study was therefore designed to determine the prevalence and predictors of self-medication with antibiotics in North-eastern Tanzania and propose appropriate interventions in mitigating the globally rising crisis of antibiotic resistance.

## Materials and methods

### Study design, site and population

This was a community based cross sectional study which was conducted in SanyaJuu ward, Siha district in Kilimanjaro region, North-eastern Tanzania with a population of 116,313[26]. Siha is one of the seven administrative districts of Kilimanjaro Region with mostly a rural mixed small business and peasant inhabitants. It covers approximately 1158 square kilometers (447 square miles) and administratively divided into 12 wards. It is bordered to the west by Arusha region and to the east by Rombo and Hai districts. The western part of Mount Kilimanjaro is located within the district boundaries. The distribution of health facilities is such that there is a district hospital, five health centres and thirteen dispensaries, minimally equipped as in many rural areas in a developing country. Pharmacies and drug outlets are evenly spread throughout the district.

### Sample size and data collection

On the basis of the prevalence of self-medication of 71.5% in Ilala district, Dar es Salaam, Tanzania[27] and a relative precision of 5% at 95% confidence interval the minimum sample size was estimated to be 313 using the Kish Lisle et al (1965)[28] formula to determine the sample size for cross—sectional studies: n = Z^2^P (1-P)/d^2^.

Out of seven districts of Kilimanjaro region Siha district was randomly selected. Sanya Juu ward was randomly selected. Systematic sampling was used to select households in all villages of Sanya Juu ward. Head of the household was selected to participate in the study or, in his absence, any adult member above 18 years present was selected. In the presence of more than one adult, always the oldest was chosen as the respondent. Individuals not willing to participate were excluded. A total of 300 respondents were interviewed. Data were collected using a semi-structured questionnaire developed by a research team.

### Validity of the questionnaire

The validity and reliability of the questionnaire were determined by using computer software IBM SPSS Version 20. To maximize its validity, the questionnaire was pre-tested on relevant respondents before data collection. Ten community members were selected. These respondents were excluded during data collection for this study. In addition two experts in the field of survey design approved the quality of the questionnaire. After the pre-test, adjustments in phrasing were made.

### Data collection

The principal investigator together with two trained research assistants administered the questionnaire from 1^st^ to 28^th^ April 2017. The questionnaire captured socio–demographics, self–medication practices with antibiotics and other associated conditions like sources of drugs used, reasons for antibiotic self- medication and type of antibiotics employed for self medication.

### Data management and analysis

Data were entered into an excel spreadsheet and STATA version 13 (StataCorp, College Station, TX, USA) was used for statistical analyses. Data were visually examined for distribution, missing values and outliers using univariable analysis. We described baseline characteristics of the study population by using descriptive statistics. Median (inter-quartile range) was used to summarize continuous variables and frequencies and proportions for categorical variables. Log-binomial regression was performed to determine the association between socio-demographic characteristics and self-medication. Crude and adjusted Odds Ratio (ORs) or prevalence ratios together with their 95% confidence intervals were estimated as measures of association. We added all variables from the bivariate log-binomial regression into a multivariate log-binomial regression model regardless of their significance. In the final model, the p-value of <0.05 was considered to be significant.

### Ethical considerations

Ethical approval to conduct this study was obtained from Kilimanjaro Christian Medical University College Ethics Committee Certificate number 892. Permission to conduct the study was obtained from the District Executive Director of Siha District and Sanya Juu Ward Executive Officer (WEO). Written informed consent to participate was obtained from all individuals who agreed to participate in the study prior to participation. All measures to protect the privacy and confidentiality were considered in that neither names nor house registration numbers were mentioned and recorded during data collection.

## Results

### Characteristics of the study participants

A total of 300 individuals participated in this study. The median (IQR) age of participants was 23 (20.5–36.5) years. The majority of the participants, 222 (74.00%) were youth and the proportion of males and females was almost similar among the study participants and comparable to the study population. More than half of the participants had attained primary education or higher and a higher proportion (94.67%) were unemployed. The prevalence of antibiotic self-medication was 58% (Table 1).

**Table 1:** Prevalence of self-medication and characteristics of study participants (N = 300).

Variable		Self–medication n (%)		
	Overall (N = 300)	Yes (n = 174)	No (n = 126)	χ2 p-value
Median age years (IQR)*	23(20.5–36.5)			
**Age groups**				
18–35	222	124 (55.9)	98 (44.1)	0.114
36–60	68	46 (67.60)	22 (32.4)	
61–78	10	04 (40.0)	06 (60.0)	
**Sex**				
Male	160	91 (56.9)	69 (43.1)	0.673
Female	140	83 (59.3)	57(40.7)	
**Marital status**				
Married	139	80 (57.6)	59 (42.4)	0.884
Unmarried	161	94 (58.4)	67 (41.6)	
**Educational level**				
No formal education	17	09 (52.9)	08 (47.1)	0.389
Primary	111	70 (63.1)	41 (36.9)	
Above primary	172	95 (55.2)	77 (44.8)	
**Occupation**				
Employed	16	13 (81.2)	03 (18.8)	0.053
Unemployed	284	161(56.7)	123 (43.3)	
**Income (TZs)**				
<180,000	287	166 (57.8)	121 (42.2)	0.792
>180,000	13	08 (61.5)	05 (38.5)	

*IQR = Interquartile range.

### Symptoms, antibiotics and reasons for self-medication

Above ninety percent of the participants reported to go to the pharmacy for care when they fall sick. The most common symptoms reported by self-medicating participants were cough (51%), fever (23%) and diarrhoea (22%). The most common self- medicating antibiotics employed were amoxicillin (43%) and an antiprotozoal drug metronidazole (10%). The most commonly reported reasons for self-medication were emergency illness (24.0%), health facility charges (20.33%), proximity of pharmacy to home place (17.00%) and no reported reason (16.66%) (Table 2).

**Table 2:** Symptoms, antibiotics and reasons for self-medication (N = 300).

Variable	n(%)
**Action on falling sick**	
Go to hospital	20 (6.67)
Go to pharmacy	276(92.00)
Treating myself at home	03 (1.00)
Others	01 (0.33)
	
**Symptoms**	
Headache/ Fever/ Malaria	45 (25.57)
Diarrhoea	38 (21.59)
Cough	90 (51.14)
Injury	3 (1.70)
	
**Antibiotics**	
Amoxicillin	130 (43.33)
Tetracycline	5 (1.67)
Chloramphenicol	3 (1.00)
Ciprofloxacin	1 (0.33)
Ampiclox	3 (1.00)
Others **Antiprotozoa**	1 (0.33)
Metronidazole	31 (10.33)
	
**Reasons**	
Emergency illness	72 (24.00)
Long distance from the health facility	38 (12.67)
Proximity of pharmacy to home place	51 (17.00)
Health facility charges	61 (20.33)
No medicine in the health facility	3 (1.00)
Delaying of hospital services	25 (8.33)
No reason given	50 (16.66)

### Factors associated with self-medication

On a bivariate log-binomial regression, being an adult (OR: 1.65; 95% CI (0.93–2.93), female (OR: 1.10; 95% CI (0.69–1.75), unmarried (OR: 1.03; 95% CI (0.65–1.63), having a primary education (OR: 1.51; 95% CI (0.54–4.23) and having an income of more than TShs180,000 (OR: 1.16; 95% CI (0.37–3.65), were associated with the increased risk of self-medication. Other factors were not statistically significant (Wald test p value > 0.05). On a multivariate log-binomial regression after adjusting for variables (age, sex, education, marital status), being an adult (OR: 1.73; 95% CI (0.86–3.50), female (OR: 1.09; 95% CI (0.80–1.79), unmarried (OR: 1.14; 95% CI (0.80–2.75), and having a primary education (OR: 1.45; 95% CI (0.46–4.51), remained to be associated with the increased risk of self—medication although the association was not statistically significant (Wald test p-value >0.05) (Table 3).

**Table 3:** Bivariate and multivariate logistic regression of factors associated with self-medication.

Variable	Total	Self-medication	CRUDE		ADJUSTED	
	n	Yes (n (%))	OR (95% CI)	p- value	OR (95% CI)	p- value
**Age group**						
18–35	222	124 (55.9)	1(baseline)		1 (baseline)	
36–60	68	46 (67.6)	1.65(0.93–2.93)	0.086	1.73(0.86–3.50)	0.126
61–78	10	04 (40.0)	0.52(0.14–1.91)	0.331	0.64(0.32–1.67)	0.554
**Sex**						
Male	160	91 (56.9)	1(baseline)		1 (baseline)	
Female	140	83 (59.3)	1.10(0.69–1.75)	0.673	1.09(0.68–1.78)	0.705
**Marital status**						
Married	139	80 (57.6)	1(baseline)		1 (baseline)	
Unmarried	161	94 (58.4)	1.03(0.65–1.63)	0.884	1.49(0.80–2.75)	0.206
**Educational level**						
No formal education	17	09 (52.9)	1(baseline)		1 (baseline)	
Primary	111	70 (63.1)	1.51(0.54–4.23)	0.426	1.45(0.46–4.51)	0.522
Above primary	172	95 (55.2)	1.09 (0.40–2.97)	0.856	1.02(0.32–3.25)	0.978
**Occupation**						
Employed	16	13 (81.2)	1(baseline)		a	a
Unemployed	284	161 (56.7)	0.30 (0.08–1.08)	0.066		
**Income (TZS†)**						
<180,000	287	166 (57.8)	1(baseline)			
>180,000	13	08 (61.5)	1.16(0.37–3.65)	0.792	a	a

†TZS = Tanzanian shillings (1 United States Dollar = 2, 230 TZS).

^a^ Multivariate estimates were too small to be interpreted and therefore omitted.

### Knowledge on self-medication

All study participants had heard about antibiotic self-medication and 98% said self-medication couldn’t be practiced with all drugs. When sick, 92% went to pharmacy for care. 98% responded that self-medication could result to harmful effects. Ninety nine percent knew that self—medication was not better than medical consultation and above 95% of the participants reported to know that self-medication could result to delays for one to seek medical care as well as development of drug resistance (Table 4).

**Table 4:** Knowledge on self–medication.

Variable	Yes	No	Don't know
	n (%)	n (%)	n (%)
Ever heard about self-medication	300(100.00)	0(0)	0(0)
Self-medication can be practiced with all drugs	06(02.00)	294(98)	0(0)
Self-medication is better than seeking med consultation	0 (0.00)	299(99.67)	1(0.33)
Same medicine can be shared between two people with different ailment	09 (03.00)	265(88.33)	26(08.66)
Self-medication practices result into harmful effects	294(98.00)	04(01.33)	02(0.67)
Self-medication causes addiction	298(99.33)	00(0.00)	02(0.67)
Self-medication delays one to seek medical care	289(96.33)	02(0.67)	09(03.00)
Self-medication results to drug resistance	289(96.33)	02(0.67)	09(03.00)
Self-medication results int complications	269(89.67)	04(01.33)	27(09.00)

## Discussion

The study aimed at determining the prevalence and factors for antibiotic self-medication in a rural area of Kilimanjaro Region. It is important to establish and quantify existence of antibiotic self-medication and associated factors due to its consequent antimicrobial resistance for control and mitigation of the vice. The prevalence of antibiotic self–medication in this population was 58%.

Our data indicate that more than half of the study population self-medicated with antibiotics. This may be due to lack of health care facilities that adequately cater for the health care needs of the community. One may resort to self-medication in a rural developing country due to non-sustainable medicine supply in health facilities, difficulties in seeing a clinician due to congestion, lack of money to pay for health services, long distance to the health facility, delaying of hospital services, ignorance of the consequences and lack of antibiotic regulatory control[29–32]. Dissatisfaction with medical care has also been documented[29,33]. Among other effects, antibiotic self–medication has been associated with development of antibiotic resistance[3,34–37].

The data show that more than nine out of ten individuals went to pharmacy for care when they fall sick. It is a common practice in developing countries to directly resort to pharmacy visits instead of medical consultation for care[38]. One of the factors identified from the data was health facility charges. High health care costs causes people avoid attending clinics thus self-medicate. This practice has deleterious effects on fostering development of antibiotic resistance. An attempt to avail health care plans, as health insurance, coupled with control of access to antibiotics and enforcement of the laws and regulations on antibiotic dispensing would mitigate this problem.

Diarrhoea was the second common symptom for self-medication. Most of the management of diarrhoea is supportive by oral rehydration and intravenous infusions[39]. Antibiotic self-medication with diarrhoea is largely inappropriate. An antibiotic is only indicated for a laboratory confirmed infective diarrhoea[39]. In Tanzania there is a habit of taking metronidazole for self-treatment of diarrhoea[23]. The 22 percent diarrhoea patients that self–medicated with antibiotics are an indication of the extent of inappropriate antibiotic use–a practice that eventually leads to development of antibiotic resistance. Mass education should be routinely implemented to raise awareness of people to employ fluid replacement as the first line of action when one contracts diarrhoea. One study showed that most diarrhoea to be of viral origin than bacterial hence in most cases antibiotics are not warranted as part of management[40].

The antibiotics mostly used for self-medication were penicillins (43%). We observe that penicillins are the most commonly known of many antibiotics and freely available, hence potential for self-medication in many countries since they are believed to treat many ailments[41–43]. It is thus not surprising that bacterial resistance against this class of antibiotics is on the rise[44,45]. More evidence of isolates of *Escherichia coli* resistant against penicillins has been published to be between 30%-81.6%[46–49]. Amoxicillin has been found in other studies to be the most self-medicated antibiotic in Kenya (36%)[42], Ghana (23.9%)[50], United Arab Emirates (46.3%)[51], Iran (40.5%)[52] and in Karachi, Pakistan (41.4%)[31,53].

The second most common drug used for self-medicated was antiprotozoal medicine metronidazole (10%). An observation on the most frequent self-medication with metronidazole occurs in treatment of diarrhoea[23]. Since diarrhoea is very common and the standard treatment is mostly supportive, this antiprotozoal drug is inappropriately consumed. Metronidazole was concomitantly the second most self-medicated antiprotozoa in another study in India[54]. However most studies have not reported a high bacterial resistance against metronidazole especially in developing countries[25,55]. The risk is therefore high for development of drug resistance against amoxycillin and metronidazole due to their excessive and inappropriate use.

In Tanzania antibiotics can be available and accessed in rural and peri-urban areas through Accredited Drug Dispensing Outlets (ADDO) Programme[56–58]. Dispensing antibiotics even through ADDO requires a prescription. The antibiotic access policy in Tanzania requires that antibiotics are strictly prescription only medicines. In order to increase access to antibiotics to the needy so that minor infections may be controlled countrywide, considering the lack of enough trained pharmacy personnel the government of Tanzania introduced a programme called Accredited Drug Dispensing Outlets (ADDO). This entails a short course training on dispensing knowledge and skills with certification after which the graduate gets employed in a drug shop where majority of such drug outlets are in rural Tanzania [56,57]. In this course, it is well emphasized that antibiotics are prescription only drugs.

The accessibility of antibiotics through ADDO initiative has occasionally negatively resulted to abuse with evidence of non-prescription purchase of antibiotics[59–61]. A more serious supportive supervision, enforcement of dispensing regulations and massive dispenser and consumer education need to be instituted by TFDA.

We found a high level of awareness of the negative consequences of antibiotic self-medication despite a reported high practice of the same. All the study population were aware of harmful effects of antibiotic self-medication including antibiotic resistance. They also reported to have heard of self-medication, but, surprisingly 98% had practiced self-medication. This is cognitive dissonance theory at work. Sixteen percent of the self-medicating respondents had no reason why they practiced it. Clearly, a deliberate and a serious government initiative to enforce the restriction of availability and inappropriate accessibility to antibiotics may not be over emphasized.

The most important reasons for antibiotic self–medication given by participants were emergency illness (24%) and health facility charges (20%). Emergency illness are taken care of at home instead of health care facility thereby encouraging self-medication mostly from “left over” antibiotics[43]. Health facility charges contribute greatly to self-medication as people try “cutting medical costs” in avoiding high cost of doctor consultation, and other economic factors like logistics to reach the health facility[41,62–64]. Relative to purchasing power of the rural mass in Tanzania majority cannot afford even the supposedly free healthcare in public health facilities. Despite subsidies effected by the government in health care provision, there are still mandatory costs that must be paid by patients seeking medical care, including transport costs, card opening, laboratory and medicine costs.

This study commands strength by involving the general population, which is an attempt to describe in practical terms a reality on the ground in self-medication practice, unlike other studies that involved institutionalized populations like university students, health-care workers or outpatients in a hospital [31,33,64–69]. The inherent limitation in this study was recall bias. Some of the participants had difficulties in remembering antibiotic names and whether or not they had self-medicated in the previous six months thus impacting on the prevalence of self-medication. However, in the current study six months recall bias was minimized by encouraging the respondents to relate important life events such as birthdays, planting season and religious festivals to illness so that a self medication event could easily be recalled. Secondly, our calculated sample size was 313 but we managed to interview 300 respondents. 13 could not be reached despite our two consecutive attempts to visit them at home. However, we believe the results of this study are important as baseline information to health care managers and policy makers to plan intervention programs related to antibiotic self-medication.

## Conclusions

Self-medication with antibiotics is practiced by more than a half of Siha district residents predominantly by adults than youth and elderly. Majority (92%) of respondents go to pharmacy for care on falling sick. Antibiotic self-medication is mostly due to cough, fever and diarrhoea. Factors associated with increased risk of antibiotic self-medication are being an unmarried adult female with a primary school education. Despite a high awareness of the negative effects of self-medication, self-medication practice remains high in Siha district. The findings of this study warrant government intervention through TFDA by mass education, dispensers’ education and lawfully enforcing restriction on access to antibiotics on prescription only policy. An avenue for further study should envisage identifying, through a qualitative methodology, whether the same respondents in this study understand the consequences of antibiotic resistance caused by antibiotic self-medication.

## Supporting information

S1 Questionnaire (DOC)
