# Supplementary material for: Prevalence, determinants and knowledge of antibacterial self-medication: A cross sectional study in North-eastern Tanzania
Source: PLoS One. 2018 Oct 31;13(10):e0206623. doi: 10.1371/journal.pone.0206623 (PMC6209340; doi:10.1371/journal.pone.0206623)
Supplement: S1 Questionnaire — (DOC) [file pone.0206623.s001.doc]

**Supplementary Information 1 (SI1)**

**QUESTIONNAIRE FOR DATA COLLECTION**

**SELF - MEDICATION WITH ANTIBACTERIALS AMONG COMMUNITY MEMBERS IN SANYA JUU WARD IN SIHA DISTRICT KILIMANJARO REGION, NORTH - EASTERN TANZANIA.**

**General information**

**Data of interview: (DD/MM/YYYY) ……../……./………**

**Participants ID No: ………………………………………………**

**Municipality………………….Ward/Village……………………**

**Street……………………………………………………………….**

| **Section one: Social-demographic information** | | | |
| --- | --- | --- | --- |
| No | Question | Response | Coding |
| 1 | Ages in years |  |  |
| 2 | Sex | (1) Male  (2) Female |  |
| 3 | Marital Status | (1) Single  (2) Married  (3) Divorced  (4) Widow  (5) Cohabiting |  |
| 4 | Education level | (1) No formal Education  (2) Primary  (3) Secondary  (4) Tertiary |  |
| 5 | Occupation | (1) Employed  (2) Unemployed  (3) Businessman/woman  (4) Student |  |
| 6 | Income (Tzs) | (1) <180,000/=  (2) Between 180,000 and 1,000,000  (3) >1,000,000/= |  |

| **Section two: Information concerning knowledge on Self-medication** | | | |
| --- | --- | --- | --- |
| 7 | Have you ever heard about self-medication? | (1) Yes  (2) No |  |
| 8 | Can self-medication be practiced with all drugs | (1) Yes  (2) No |  |
| 9 | When you get sick what do you do? | (1) Go to hospital  (2) Go to pharmacy  (3) Go to traditional healer  (4) Treating myself at home  (5) Others ………………… |  |
| 10 | Is self-medication better than seeking medical consultation? | (1) Yes  (2) No  (3) I don’t know |  |
| 11 | Can the same medicine be shared between two people having different ailment? | (1) Yes  (2) No  (3) I don’t know |  |
| 12 | Can self-medication practices result into harmful effect? | (1) Yes  (2) No  (3) I don’t know |  |
| 13 | Can self-medication cause addiction? | (1) Yes  (2) No  (3) I don’t know |  |
| 14 | Can self-medication delay one to seek for hospital intervention | (1) Yes  (2) No  (3) I don’t know |  |
| 15 | Can self-medication result to drug resistance | (1) Yes  (2) No  (3) I don’t know |  |
| 16 | Can self-medication result into complication like new illness | (1) Yes  (2) No  (3) I don’t know |  |

| **Section three: Information of self-medication practices** | | | |
| --- | --- | --- | --- |
| 17 | Have you fallen sick for the past six months? | (1) Yes  (2) No  (3) I don’t remember |  |
| 18 | What was/were the illness that you was suffering from | (1) Headache  (2) Diarrhea  (3) Malaria  (4) Fever  (5) Coughing  (6) Eye infection  (7) Skin infection  (8) Injury  (9) Other…………. |  |
| 19 | Did you self-medicate with any antibiotics in the last six months? | (1) Yes  (2) No |  |
| 20 | What antibiotic(s) did you use for self-medication? (can be more than one) | (1) Amoxicillin  (2) Doxycycline  (3) Tetracycline  (4) Erythromycin  (5) Chloramphenicol  (6) Metronidazole  (7) Ciprofloxacin  (9) Other………………… |  |
| 21 | How often have you been taking the antibiotic (s) without a prescription | (1) Never  (2) Once  (3) Less often (occasionally)  (4) More often (frequently)  (5) More times I can’t even remember |  |
| 22 | Who prescribed the medication (s) for you? | (1) Medical personnel from health facility  (2) Worker in the pharmacy  (3) A friend  (4) None |  |
| 23 | Have you ever given medication to another person without prescription? | (1) Yes  (2) No |  |
| 24 | If “yes” how often have you been giving the medication (s) without a prescription? | (1) Frequently  (2) Occasionally  (3) Rarely |  |
| 25 | What was the source (s) of medicine which you have been using? | (1) Government health institution  (2) Bought from a pharmacy  (3) Got from a relative or friend/relative  (4) Left over from previous illness  (5) Information from the internet  (6) Read from the newspaper  (7) Heard from radio  (8) Others………………………… |  |
| 26 | Were you told about the instructions on how the person should be given the medications? | (1) Yes  (2) No |  |
| 27 | What was the outcome of the medicine which have used? | (1) Recovered  (2) Improved  (3) Did not improve  (4) Worsened |  |
| 28 | If worsened, what else did you do? | (1) Went to the hospital  (2) Went to buy more drugs  (3) Went to traditional healer  (4) Just stayed at home |  |
| 29 | What did you use medication (s) without prescription instead of going to health facility? | (1) Emergency illness  (2) Distance to the health facility  (3) Proximity of the pharmacy to home place  (4) Health facility charges  (5) No medicine in health facilities  (6) Delaying of the hospital services  (7) Others ……………………… |  |
| 30 | Did you observe any problems after self-medicating with antibiotics? | (1) Yes  (2) No |  |
| 31 | If “yes” what problem did you observe after self-medication with antibiotics? | (1) Body rash  (2) Swollen face  (3) Yellowish eyes  (4) Severe vomiting blood  (5) Severe diarrhea  (6) Condition worsened  (7) Others………………….. |  |

**End of interview:** Thank you for your cooperation and your time for accepting to participate in this study and may God bless you.

**Local Language: Kiswahili version of the questionnaire**

**DODOSO KWA AJILI YA UKUSANYAJI WA TAARIFA**

**UTAFITI KUHUSU UTUMIAJI WA DAWA TIBA ZA VIUAVIJASUMU KWA WANANCHI WA KATA YA SANYA JUU ILIYOPO WILAYA YA SIHA KATIKA MKOA WA KILIMANJARO, KASKAZINI MASHARIKI MWA TANZANIA.**

**Taarifa kwa ujumla**

**Tarehe ya mahojiano: ……/….. /2017**

**Namba ya kitambulisho cha mshiriki: …………………………**

**Manispaa ya: ………………………. Kata/ Kijij: ……………………………...**

**Mtaa: ……………………………**

| **Sehemu ya kwanza: Taarifa kuhusu anayehojiwa** | | | |
| --- | --- | --- | --- |
| Nambari | Swali | Majibu | Namba ya jibu |
| 1 | Umri katika miaka | ………… |  |
| 2 | Jinsia | (1) Mwanaume  (2) Mwanamke |  |
| 3 | Hali ya ndoa | (1) Hajaoa/olewa  (2) Ndoa  (3) Talaka  (4) Mwanamke mjane  (5) Kimada |  |
| 4 | Kiwango cha elimu | (1) Hajawahi kusoma  (2) Elimu ya msingi  (3) Elimu ya sekondari kidato cha nne  (4) Elimu zaidi ya kidato cha nne  (5) Elimu ya juu (chuo kikuu) |  |
| 5 | Kazi | (1) Muajiriwa  (2) Hajaajiriwa  (3) Mfanyabiashara wa kiume/kike  (4) Mwanafunzi |  |
| 6 | Kipato [Shilingi za Tanzania (Tshs)] | (1) Chini ya 180,000/=  (2) Kati ya 180,000 na 1,000,000/=  (3) Zaidi ya 1,000,000/= |  |

| **Sehemu ya pili: Taarifa kuhusu ufahamu juu ya utumiaji wa dawa pasipo ushauri wa kitaalamu** | | | |
| --- | --- | --- | --- |
| 7 | Ulishawahi kusikia kuhusu utumiaji wa dawa tiba pasipo ushauri wa kitaalamu? | (1) Ndio  (2) Hapana |  |
| 8 | Je, utumiaji wa dawa tiba pasipo ushauri wa kitaalamu unaweza kufanyika kwa dawa zote? | (1) Ndio  (2) Hapana  (3) Sijui |  |
| 9 | Wakati  Unapo ugua unafanya nini? | (1) Nakwenda hospitali  (2) Naenda famasi  (3) Naendakwamtaalamuwatibaasilia  (4) Najitibu mwenyewe nyumbani  (5) Mengineyo…………………… |  |
| 10 | Je, utumiaji wa dawa tiba pasipo ushauri wa kitaalamu ni bora kuliko kwenda hospitali? | (1) Ndio  (2) Hapana  (3) Sijui |  |
| 11 | Je, dawa moja inaweza kutumika na watu wawili wenye magonjwa tofauti? | (1) Ndio  (2) Hapana  (3) Sijui |  |
| 12 | Je, kutumia dawa tiba pasipo ushauri wa kitaalamu kunaweza kusababisha madhara? | (1) Ndio  (2) Hapana  (3) Sijui |  |
| 13 | Je, kutumia dawa tiba pasipo ushauri wa kitaalamu kunaweza kusababisha mtu kuzizoea vibaya? | (1) Ndio  (2) Hapana  (3) Sijui |  |
| 14 | Je kutumia dawa tiba pasipo ushauri wa kitaalamu kunaweza kuchelewesha mtu kutafuta msaada hospitalini na hatimaye kuzidiwa? | (1) Ndio  (2) Hapana  (3) Sijui |  |
| 15 | Je, kutumia dawa tiba pasipo ushauri wa kitaalamu kunaweza kusababisha dawa kuwa sugu kutibu? | (1) Ndio  (2) Hapana  (3) Sijui |  |
| 16 | Je, kutumia dawa tiba pasipo ushauri wa kitaalamu kunaweza kusababisha magonjwa maengine? | (1) Ndio  (2) Hapana  (3) Sijui |  |

| **Sehemu ya tatu: Taarifa kuhusu utumiaji dawa tiba pasipo ushauri wa kitaalamu** | | | |
| --- | --- | --- | --- |
| 17 | Je, kwa kipindi cha miezi sita, umewahi kuugua? | (1) Ndio  (2) Hapana  (3) Sikumbuki |  |
| 18 | Kama ndio, Je, uliugua kipi kati ya magonjwa yafuatayo? | (1) Maumivu ya kichwa  (2) Kuhara  (3) Malaria  (4) Homa  (5) Kukohoa  (6) Ugonjwa wa macho  (7) Ugonjwa wa ngozi  (8) Jeraha  (9) Mengineyo…………………….. |  |
| 19 | Je, ulitumia dawa? | (1) Ndiyo  (2) Hapana |  |
| 20 | Je, ni dawa tiba ulitumia kati ya hizi (unaweza kutaja zaidi ya moja) | (1) Amoxicillin  (2) Doxycycline  (3) Tetracycline  (4) Erythromycin  (5) Chloramphenicol  (6) Metronidazole  (7) Ciprofloxacin  (8) Mengineyo………………… |  |
| 21 | Je, ni mara ngapi umetumia dawa bila ushauri wa kitaalamu? | (1) Sijawahi  (2) Mara moja  (3) Mara chache  (4) Mara nyingi  (5) Mara nyingi hata sikumbuki |  |
| 22 | Ulipata ushauri wa dawa tiba kutoka kwa nani? | (1) Mtaalamu wa hospital/zahanati  (2) Mtaalamu kutoka maduka ya dawa  (3) Kutoka kwa rafiki  (4) Hakuna aliyenishauri |  |
| 23 | Umewahi kumpa mtu yeyote hapa nyumbani bila ushauri wa kitaalamu? | (1) Ndiyo  (2) Hapana |  |
| 24 | Kama ndiyo, je, ni mara ngapi umekuwa ukiwapa watu dawa tiba bila ushauri wa kitaalamu? | (1) Mara nyingi  (1) Mara chache  (3) Kwa nadra sana |  |
| 25 | Ni nini chanzo/nyanzo vya dawa tiba ulizotumia au umekuwa ukitumia? | (1) Kutoka kituo cha afya  (2) Kununuliwa kutoka duka la dawa  (3) Kutoa kwa ndugu/rafiki  (4) Zilizobaki kutoka ugonjwa uliopita  (5) Taarifa kutoka kwenye mitandao  (6) Kusoma kwenye magazeti  (7) Kusikiliza kwenye redio  (8) Mengineyo……………………… |  |
| 26 | Je, ulielekezwa kuhusu utumiaji wa dawa hizo? | (1) Ndiyo  (2) Hapana |  |
| 27 | Ni nini matokeo ya dawa tiba ulizotumia au kuwapa wengine bila ushauri wa kitaalamu? | (1) Kupona  (2) Kupata nafuu  (3) Hazikunisaidia  (4) Kuzidiwa |  |
| 28 | Kama ulizidiwa ni nini kingine ulifanya | (1) Kwenda hospitali  (2) Kwenda kununua dawa zaidi  (3) Kwenda kwa mganga wa jadi  (4) Ulibaki nyumbani  (5) Mengineyo……………… |  |
| 29 | Ni nini kilicho kufanya kutumia dawa tiba pasipo ushauri wa kitaalamu badala ya kwenda hospitalini? | (1) Dharura ya ugonjwa  (2) Umbali wa kituo cha afya  (3) Ukaribu wa maduka ya dawa  (4) Gharama za kwenye kituo cha afya  (5) Hakuna dawa katika kituo cha afya  (6) Inachukua muda mrefu kupata matibabu  (7) Mengineyo……………………. |  |
| 30 | Je, ulishawahi kupata matatizo yeyote baada ya kutumia dawa tiba bila ya ushauri wa kitaalamu? | (1) Ndiyo  (2) Hapana |  |
| 31 | Kama ndio je, ni tatizo gani lilitokea? | (1) Upele mwilini  (2) Kuvimba uso  (3) Macho ya njano  (4) Kutapika damu sana  (5) Kuharisha sana  (6) Kutapika sana  (7) Hali ilikuwa mbaya sana  (8) Mengineyo……………………. |  |

**Mwisho wa mahojiano**: Asante sana kwa ushirikiano wako, muda wako na kukubali kushiriki katika huu utafiti, Mungu akubariki.
